# Domestic Summary

**Published:** 1838-03-14

**Authors:** 


### DOMESTIC SUMMARY.

*A new treatment in a case of Anchylosis.* By J. Rhea Barton, M. D.

The February number of the *American Journal of the Medical Sciences*, contains an interesting article on a new method of treating anchylosis, by Dr. J. Rhea Barton. We subjoin a brief summary of the case. The patient when nine years of age, had a violent attack of inflammation in the knee joint, which occasioned the "destruction of the synovial membranes, the ligaments, cartilages, and, in short, everystructure appertaining to the joint," and terminated in true anchylosis, the bones of the leg and thigh having become united.

"The loss of the articulation of the knee, did not constitute the sadness of the case. It was caused by mal-position of the limb; the leg having been flexed upon the thigh to a degree somewhat less than a right angle." In this condition the patient suffered for sixteen years, when he repaired to this city, and on the 27th of May, 1835, the following operation was performed for his relief. "Two incisions were made over the femur, just above the patella. The first commenced at a point opposite the upper and anterior margin of the external condyle of the femur, and, passing obliquely across the front of the thigh, terminated on the inner side. The second incision commenced also on the outer side, about two and a half inches above the first, and passing likewise obliquely across the thigh, terminated with the other at an acute angle. By these incisions were divided the integuments, the tendon of the extensor muscles of the leg, at the insertion into the upper part of the patella, and some of the contiguous fibres of the rectus and crureus muscles themselves, a greater part of the vastus internus, and a portion of the vastus externus muscles. A flap, composed therefore of this structure, was elevated from the femur close to the condyles. The soft parts were next detached from the outer side of the bone, from the base of the flap towards the ham, by passing a knife over the circumference of it, so as to admit of the use of a saw. The flap being turned aside, a triangular or wedge-like piece of the femur was easily removed by means of a small narrow bladed saw. This wedge of bone did not include the entire diameter of the femur at the point of section; so that a few lines of the posterior portion of the shaft of the bone remained yet undivided. By slightly inclining the leg backward these yielded, and the solution was complete. This mode of effecting the lesion of the bone was designedly adopted, and constituted what I conceived to be a very important measure in the operation. Important, because it rendered the popliteal artery free from the danger of being wounded by the action of the saw, and subsequently the interlocking of the fractured surfaces tended to retain the extremities of the divided bone in their positions until the harshness of their surfaces had been overcome either by the absorption of their angles, or by the deposition of new matter upon them—a change essential to the safety of the artery during the subsequent treatment of the case. Not a blood vessel was opened which required either ligature or compression. The operation, which lasted about five minutes, being thus ended, the reflected flap was restored to its place, the wound lightly dressed, and the patient put to bed, lying on his back, with his limb supported on a splint of *an angle corresponding to that of the knee previous to the operation.* This position was maintained until it was believed that the asperities of the bone had become blunted, and were not likely by their pressure to cause ulceration of the artery beneath them. This first splint was then removed, and another having the angle slightly obtuse, was substituted. In a few days a third splint, with the angle more obtuse than that of the second, supplied its place. Others varying in degrees of angularity, in like manner came in their turn to support the limb until it had obtained a position almost straight. It was then unchangeably continued in that line until the contact surfaces of the bone had united and securely fixed the limb in this the desired direction. During the treatment of the case, especial care was bestowed in protecting the popliteal vessels against any injurious encroachment upon them; with that view, all antagonising pressure on the soft parts in the ham was carefully avoided. The limb was rested on two long bran bags laid upon the splint, with their ends apart, a vacancy of four or five inches being left between them opposite the lesion of the bone. This inter space was lightly filled with carded cotton, so as to afford a safe support."

The constitutional symptoms which followed the operation were those commonly attendant upon compound fracture, but were never alarming. The cure occupied four months. The straightening of the limb was accomplished in two, and complete consolidation of the bone did not take place until the end of the fourth.

The report of the operation was designedly delayed, in order that the result might be entirely satisfactory. A letter dated November 6th, 1837, written by the patient is appended to the case, in which he tells us that the "operation has been *completely successful.*" The patient experiences no inconvenience, except from a stiff joint, walks without stick or other aid, with the sole of the foot to the ground, and with but a slight limp. The leg and foot have increased in size, and are nearly equal to their fellows.

---

*Medical Schools.*—From catalogues lately issued, we note the number of matriculants in various Medical Schools, for the present sessions, to be as follows: 380, in the University of Pennsylvania; 227, in the Lexington Transylvania University; 122, in the Cincinnati Medical College; 100, in the Louisville Medical Institute; 82, in the Harvard University; and 80, in the Medical College of Ohio. From a report lately published, this latter institution seems to be in a prosperous condition.

---

*Mortality in Philadelphia for* 1837.—The deaths in the City and Liberties of Philadelphia, during the year 1837, were 5202, of whom 2755 were males, and 2447 females. There are reported as having died from consumption of the lungs 748, nearly one sixth of the whole number. Allowing for inaccuracy of report, and the probability that many diseases of the lungs not tuberculous are included under this head, the preponderance is still overwhelming, when we see the next highest in the list, convulsions, only 294. From scarlet fever 205 deaths occurred, from small pox 79, from measles 49, from varioloid only 2.

